# Bevacizumab with 5-fluorouracil, leucovorin, and oxaliplatin versus bevacizumab with capecitabine and oxaliplatin for metastatic colorectal carcinoma: results of a large registry-based cohort analysis

**DOI:** 10.1186/1471-2407-14-323

**Published:** 2014-05-07

**Authors:** Tomas Buchler, Tomas Pavlik, Bohuslav Melichar, Zbynek Bortlicek, Zuzana Usiakova, Ladislav Dusek, Igor Kiss, Milan Kohoutek, Vera Benesova, Rostislav Vyzula, Jitka Abrahamova, Radka Obermannova

**Affiliations:** 1 Department of Oncology and First Faculty of Medicine, Charles University and Thomayer Hospital, Videnska 800, Prague 140 59, Czech Republic; 2 Institute of Biostatistics and Analyses, Masaryk University, Kamenice 126/3, Brno 625 00, Czech Republic; 3 Department of Oncology, Palacky University Medical School and Teaching Hospital, I.P.Pavlova 6, Olomouc 775 20, Czech Republic; 4 Department of Oncology and First Faculty of Medicine, Charles University and General University Hospital, U nemocnice 2, Prague 128 08, Czech Republic; 5 Department of Comprehensive Cancer Care, Masaryk Memorial Cancer Institute, Zluty kopec 7, Brno 656 53, Czech Republic; 6 Centre for Oncology, Tomas Bata Hospital, Havlickovo nabrezi 600, Zlin 762 75, Czech Republic; 7 Department of Oncology, Jihlava Hospital, Vrchlickeho 59, Jihlava 586 33, Czech Republic

**Keywords:** Colorectal cancer, Bevacizumab, Capecitabine, 5-fluorouracil, Oxaliplatin

## Abstract

**Background:**

Data from the Czech national registry were analysed retrospectively to describe treatment outcomes for capecitabine and oxaliplatin (XELOX) regimen with bevacizumab versus 5-fluorouracil, leucovorin, and oxaliplatin (FOLFOX) regimen with bevacizumab in the first-line therapy for metastatic colorectal cancer (mCRC).

**Methods:**

A national registry containing anonymised individual data on patients treated with targeted therapies was used as a data source. In total, 2,191 mCRC patients who received a first-line therapy with bevacizumab combined with either FOLFOX regimen (n = 1,218, 55.6%) or XELOX regimen (n = 973, 44.4%) were included in the present analysis.

**Results:**

No statistically significant difference in survival was observed between the two groups, with median overall survival (OS) of 27.0 months (95% confidence interval [CI] 24.6-29.5 months) and 30.6 months (95% CI 27.8-33.4 months) for FOLFOX/bevacizumab and XELOX/bevacizumab, respectively (p = 0.281). Median progression-free survival (PFS) was 11.4 months (95% CI 10.7-12.1 months) for FOLFOX/bevacizumab and 11.5 months (95% CI 10.8-12.3 months) for XELOX/bevacizumab (p = 0.337). The number of metastatic sites was identified as the most significant predictor of PFS and, together with the presence/absence of metastatic disease at diagnosis, also for OS.

**Conclusions:**

According to this large registry-based analysis, XELOX and FOLFOX regimens have similar effectiveness for use in combination with bevacizumab in the first-line treatment of mCRC. Multiple metastatic sites and the presence of metastatic disease at diagnosis were the strongest negative predictors of OS regardless of backbone chemotherapy regimen.

## Background

Bevacizumab, a monoclonal antibody against vascular endothelial growth factor (VEGF), is currently an important component of standard therapeutic regimens for metastatic colorectal cancer (mCRC). A randomised trial has demonstrated the efficacy of bevacizumab in combination with irinotecan, bolus 5-fluorouracil (5FU), and leucovorin (IFL) [1]. The combinations of capecitabine and oxaliplatin (XELOX) and infusional 5FU, leucovorin, and oxaliplatin (FOLFOX) with bevacizumab are widely used in clinical practice as the first line treatment for mCRC, although the benefit of adding bevacizumab to FOLFOX or XELOX was smaller in the NO16966 randomised trial than that reported for the IFL regimen [2]. In addition, no differences in progression-free survival (PFS) or overall survival (OS) were observed in a phase III study of patients treated with the 5FU, leucovorin, and irinotecan with or without bevacizumab in the first line [3]. Nevertheless, bevacizumab significantly prolonged both OS and PFS when added to FOLFOX in the E3200 randomised trial enrolling patients pretreated with a fluoropyrimidine and irinotecan [4]. Adding more uncertainty about the role of bevacizumab combined with fluoropyrimidine/oxaliplatin chemotherapy, an unplanned analysis of the NO16966 study has suggested that FOLFOX/bevacizumab is not superior to FOLFOX alone although XELOX/bevacizumab was superior to XELOX alone [5].

The aim of the present registry-based study was to explore possible differences in outcomes of patients treated with bevacizumab and either XELOX or FOLFOX using data from the Czech national registry of mCRC patients containing 2,191 individual entries of patients treated with XELOX/bevacizumab or FOLFOX/bevacizumab combination for mCRC in the first line.

## Methods

### Patient database

The clinical registry CORECT (http://corect.registry.cz/) is a non-interventional post-registration database of epidemiological and clinical data of patients with mCRC treated with targeted therapies including bevacizumab, cetuximab, and panitumumab in the Czech Republic. In the Czech Republic the administration of targeted therapy outside of clinical trials is limited to comprehensive cancer centres and these drugs are reimbursed only when administered in one of these centres. The CORECT registry was created in 2011 by merging individual registries for targeted agents used in mCRC, including bevacizumab, cetuximab, and panitumumab. The registry contains anonymised individual patient data including demographic parameters, initial staging and disease characteristics, baseline patient information at the start of targeted therapy, and data on survival and adverse events. Data are entered into the database by all Czech comprehensive cancer centres administering targeted therapy and updated at least twice yearly for patients who continue treatment with targeted agents.

The study has been carried out in compliance with the Helsinki declaration and the registry has been approved by institutional ethical committees of the participating comprehensive cancer centres (the list of the centres can be found at http://corect.registry.cz/index-en.php?pg=participating-centres).

### Patients and treatment

Patients who received first-line therapy for mCRC with bevacizumab and either FOLFOX or XELOX were included in the present analysis. FOLFOX4 regimen is the predominant schedule used in most Czech centres (oxaliplatin 85 mg/m^2^ intravenously [i.v.] on day 1, leucovorin 200 mg/m^2^ i.v. on days 1 and 2, 5FU 400 mg/m^2^ i.v. bolus od days 1 and 2, and 5FU 600 mg/m^2^ 22-hour i.v. infusion on day 1, 14-day cycle) and was administered with bevacizumab 5 mg/kg i.v. on day 1 or 3 of each cycle (Saltz *et al.*[2]). However, FOLFOX6 and FOLFOX7 regimens have been used in some centres. XELOX (capecitabine 1000 mg/m^2^ twice daily orally on days 1–14, oxaliplatin 130 mg/m^2^ i.v. on day 1, 21-day cycle) was administered with bevacizumab 7.5 mg/kg on day 1 of each cycle [2]. Disease responses were assessed using the RECIST 1.1 criteria. Dose modifications were at the discretion of attending oncologist. To ensure adequate follow-up, only patients who started bevacizumab and chemotherapy at least six months prior to the data cut-off (regardless of the number of received treatment cycles) were included in the present analysis Query systems were in place for reported clinically significant toxicities.

### Statistical analysis

Standard descriptive statistics were used to describe the data. Differences in initial categorical parameters were assessed using the Fisher exact test; the Pearson chi-square test was applied when there were more than two categories. Comparisons of the treatment groups for continuous variables were based on the Mann–Whitney test. Both overall survival (OS) and progression-free survival (PFS) were calculated since the start of the bevacizumab-containing regimen. The survival was estimated using the Kaplan–Meier method. Log-rank test was used to compare OS and PFS. Multivariable Cox proportional hazards model was used to quantify the influence of the considered treatment modalities on survival in the presence of other potential predictive and prognostic factors. Model optimisation was performed via analysis of deviance and model residuals. The standard level of statistical significance at α = 0.05 was used. Differences in the occurrence of adverse effects between the two chemotherapy regimens were analysed using the Fisher exact test.

## Results

### Patient cohort

The CORECT registry included data of 4,024 mCRC patients from Czech comprehensive cancer centres who started treatment with bevacizumab between December 2005 and March 2012. Most patients (n = 3,964, 98.5%) initially received bevacizumab in combination with chemotherapy. In total, 2,191 mCRC patients (54.4% of all patients treated with bevacizumab during that period) who received a first-line therapy with bevacizumab combined with either FOLFOX regimen (n = 1,218, 55.6%) or XELOX regimen (n = 973, 44.4%) and had evaluable data as defined above were included in the present analysis. Baseline patient characteristics are shown in Table 1. As of 31 March 2012, the median follow-up was 15.9 months (range 0.1-74.0 months), with 167 (13.7%) and 133 (14.2%) patients remaining on FOLFOX/bevacizumab or XELOX/bevacizumab, respectively.

**Table 1:** Baseline characteristics of patients

**Characteristic**	**FOLFOX (n = 1218)**	**XELOX (n = 973)**	**p-value**^**1**^
**Males**, n (%)	755 (62.0)	641 (65.9)	0.060
**Age at treatment initiation**			0.859
Median, (5%-95%)	61 (43–73)	62 (43–73)	
**Localization**, n (%)			0.235
Colon	717 (58.9)	596 (61.3)	
Rectum	500 (41.1)	377 (38.7)	
Not available	1 (0.1)	0 (0.0)	
**Thromboembolism**, n (%)	44 (3.6)	36 (3.7)	0.923
**Hypertension**, n (%)	473 (40.4)	357 (39.0)	0.511
**Primarily metastatic**, n (%)			0.021*
M0	399 (32.8)	365 (37.5)	
M1	819 (67.2)	608 (62.5)	
**Histological type**, n (%)			0.123
Adenocarcinoma	1177 (96.6)	927 (95.3)	
Other	22 (1.8)	18 (1.8)	
Not available	19 (1.6)	28 (2.9)	
**PS**, n (%)			<0.001*
0	293 (24.1)	255 (26.2)	
1	303 (24.9)	130 (13.4)	
2	46 (3.8)	14 (1.4)	
3	2 (0.1)	0 (0.0)	
4	1 (0.1)	0 (0.0)	
Not available	573 (47.0)	574 (59.0)	
**Radiotherapy**, n (%)			0.235
Adjuvant	84 (6.9)	84 (8.6)	
Neo-adjuvant	112 (9.2)	112 (11.5)	
Other	18 (1.5)	14 (1.4)	
No radiotherapy	998 (81.9)	758 (77.9)	
Not available	5 (0.4)	5 (0.5)	
**Neo-adjuvant therapy**, n (%)	99 (8.2)	89 (9.2)	0.396
**Adjuvant therapy**, n (%)	288 (23.7)	262 (27.1)	0.068

*Statistically significant difference.

The best treatment responses during the first-line therapy with FOLFOX/bevacizumab or XELOX/bevacizumab, respectively, were as follows: complete response 176 (14.4%) versus 129 (13.3%); partial response 397 (32.6%) versus 303 (31.1%); stable disease 383 (31.4%) versus 394 (40.5%); progressive disease 183 (15.0%) versus 63 (6.5%) (p < 0.001). Best response was not evaluable for 79 (6.5%) and 84 (8.7%) patients, respectively.

**Figure 1:**
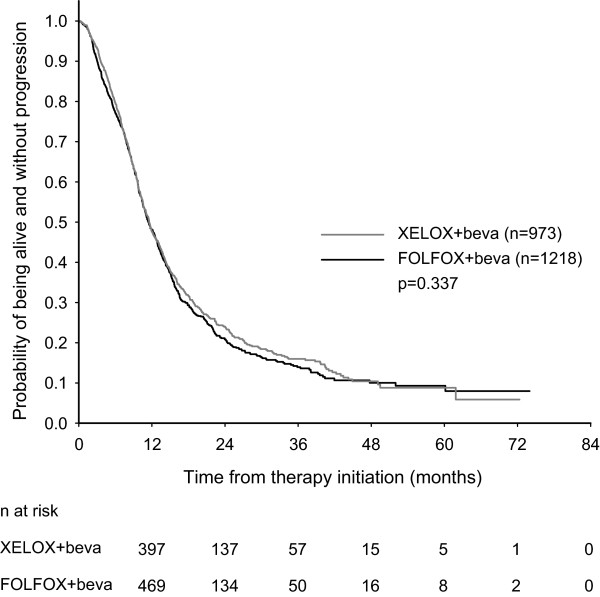
Progression-free survival of mCRC patients treated with bevacizumab in combination with FOLFOX or XELOX.

**Figure 2:**
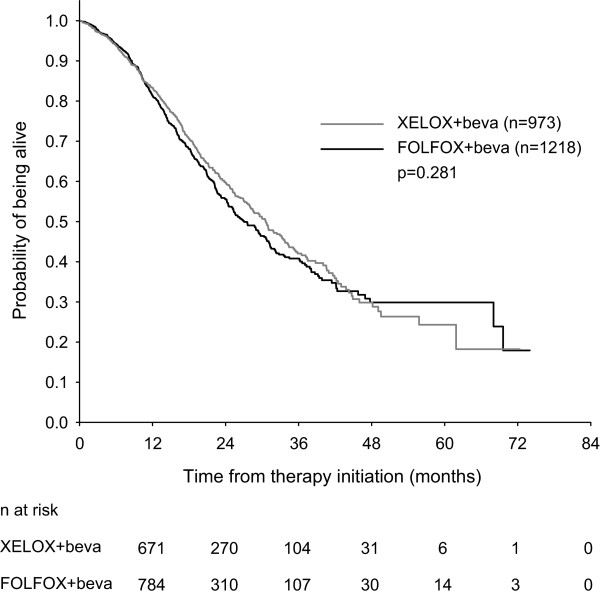
Overall survival of mCRC patients treated with bevacizumab in combination with FOLFOX or XELOX.

Median PFS was 11.4 months (95% confidence interval [CI] 10.7-12.1 months) for patients receiving bevacizumab and FOLFOX and 11.5 months (95% CI 10.8-12.3 months) for patients treated with bevacizumab and XELOX (Figure 1). This difference was not statistically significant (p = 0.337). Median OS was 27.0 months (95% CI 24.6-29.5 months) for patients receiving bevacizumab and FOLFOX and 30.6 months (95% CI 27.8-33.4 months) for patients treated with bevacizumab and XELOX (Figure 2). No statistically significant difference in OS was observed between the two treatment groups (p = 0.281).

To adjust for the influence of other potential predictive and prognostic factors, multivariable Cox model for both PFS (Table 2) and OS (Table 3) was performed. The chemotherapy regimen was not significantly associated with PFS (HR = 0.95, p = 0.400). Although sex, age, and site of primary tumour were not found to be associated with PFS, they were left in the final multivariable model as adjusting variables. The number of metastatic sites was identified as the most significant predictor of PFS (two metastatic sites: HR = 1.39, p < 0.001; three and more metastatic sites: HR = 1.66, p < 0.001).

**Table 2:** Results of the multivariable cox model for progression-free survival

**Variable**	**Risk category**	**Beta**	**HR**	**95% CI**	**p-value**
Sex	Male/female	−0.06	0.95	(0.85-1.05)	0.310
Age	>65 years/<65 years	−0.03	0.97	(0.87-1.08)	0.580
Site of primary tumour	Rectum/colon	0.08	1.08	(0.97-1.20)	0.170
Primarily metastatic	M1/M0	0.11	1.12	(1.00-1.25)	0.057
Number of metastatic sites	2/1	0.33	1.39	(1.24-1.56)	<0.001
	3 and more/1	0.51	1.66	(1.41-1.95)	<0.001
CT regimen	XELOX/FOLFOX	−0.05	0.95	(0.84-1.08)	0.400

**Table 3:** Results of the multivariable Cox model for overall survival

**Variable**	**Risk category**	**Beta**	**HR**	**95% CI**	**p-value**
Sex	Male/female	0.00	1.00	(0.86-1.15)	0.950
Age	>65 years/<65 years	−0.03	0.97	(0.83-1.13)	0.680
Site of primary tumour	Rectum/colon	0.11	1.12	(0.97-1.29)	0.130
Primarily metastatic	M1/M0	0.29	1.34	(1.14-1.56)	<0.001
Number of metastatic sites	2/1	0.38	1.47	(1.26-1.71)	<0.001
	3 and more/1	0.66	1.94	(1.57-2.39)	<0.001
Chemotherapy regimen	XELOX/FOLFOX	−0.10	0.91	(0.76-1.09)	0.290

Moreover, the multivariable Cox model for PFS was also applied on the subset of patients with available information on the performance status (n = 1,044). As in the whole dataset, the chemotherapy regimen was not found to have significant effect on PFS (HR = 1.03, p = 0.730), whereas the number of metastatic sites was confirmed as the strongest prognostic factor with respect to PFS. The performance status at the onset of targeted therapy was not found to be significantly associated with PFS. However, almost all (94.0%) patients with available performance status information in our cohort had a performance status of 0 or 1.

The results of the multivariable Cox model for OS are shown in Table 3. As for PFS, the chemotherapy regimen was not associated with OS (HR = 0.91, p = 0.290). Metastatic disease at presentation (synchronous metastases) and the number of metastatic sites were the strongest predictors of OS.

A univariate analysis has been carried out for different subgroups of patients. No particular group of patients could be identified that would profit more from FOLFOX versus XELOX backbone chemotherapy or vice versa (Table 4). Of note, wild-type KRAS oncogene status was associated with a trend to improved OS in the XELOX/bevacizumab cohort (38.8 months versus 28.4 for KRAS wild-type versus mutated, respectively; p = 0.056) but was not prognostic in the FOLFOX/bevacizumab cohort (31.3 months versus 31.5 months, respectively; p = 0.53).

**Table 4:** Overall survival and progression-free survival in different subgroups of mCRC patients

**Patient subgroup**	**First-line regimen**	**n**	**Overall survival**			**Progression-free survival**		
			**Median survival (months)**	**95% CI**	**p**	**Median survival (months)**	**95% CI**	**p**
Stage I-III at diagnosis	FOLFOX	399	31.3	26.8-39.8	0.845	12.6	11.1-14.1	0.810
	XELOX	365	33.2	28.7-41.9		12.1	11.1-13.7	
Stage IV at diagnosis	FOLFOX	819	25.3	22.7-29.5	0.311	11.1	10.5-12.1	0.370
	XELOX	608	28.5	25.1-32.9		11.2	10.1-12.2	
Without adjuvant chemotherapy	FOLFOX	927	25.2	22.5-28.8	0.212	11.1	10.5-11.9	0.414
	XELOX	704	28.7	25.5-31.8		11.2	10.5-12.1	
With adjuvant chemotherapy	FOLFOX	288	36.2	29.0-44.4	0.744	13.1	11.2-14.8	0.883
	XELOX	262	35.8	28.2-43.8		12.1	11.1-14.0	
Wild type KRAS	FOLFOX	368	31.3	27.5-37.8	0.092	11.1	10.1-12.5	0.592
	XELOX	199	38.8	34.2-45.8		10.8	9.8-12.4	
Mutant KRAS	FOLFOX	230	31.5	28.9-37.7	0.309	12.7	11.7-14.5	0.084
	XELOX	123	28.4	22.9-42.2		10.0	9.3-11.9	

### Treatment toxicity

Only toxicities considered to be related to bevacizumab were reported to the database. Safety data are summarised in Table 5. Significant (i.e. grade 3–5) adverse events were rarely reported. As expected, the most common significant adverse event irrespective of the chemotherapy regimen was hypertension. Bleeding occurred more frequently in the FOLFOX/bevacizumab cohort while diarrhoea was reported more frequently during XELOX/bevacizumab treatment. Three cases of gastrointestinal perforation were reported, all occurring in the FOLFOX/bevacizumab cohort.

**Table 5:** Incidence of significant (i.e. grade 3, 4, or 5) bevacizumab-related adverse events

	**n = 2191**	**n = 973**	**n = 1218**	
	**Total**	**XELOX**	**FOLFOX**	**p-value**
Hypertension	28 (1.3%)	15 (1.5%)	13 (1.1%)	0.344
Bleeding	10 (0.5%)	1 (0.1%)	9 (0.7%)	0.050
Gastrointestinal perforation	3 (0.1%)	0 (0.0%)	3 (0.2%)	0.259
Arterial thromboembolic event	8 (0.4%)	4 (0.4%)	4 (0.3%)	0.739
Venous thromboembolic event	17 (0.8%)	11 (1.1%)	6 (0.5%)	0.139
Proteinuria	5 (0.2%)	2 (0.2%)	3 (0.2%)	1.000

## Discussion

The present registry-based retrospective analysis suggests that the combination of bevacizumab with XELOX had similar efficacy as an infusional regimen combining bevacizumab with FOLFOX. This result is in agreement with published results of randomised clinical trials that demonstrated comparable efficacies of XELOX and FOLFOX) alone or in combination with bevacizumab [2,6,7]. In addition, three registry-based studies examining bevacizumab efficacy in mCRC have been published, including the prospective Bevacizumab Expanded Access Trial (BEAT) study, the prospective Bevacizumab Regimens’ Investigation of Treatment Effects (BRiTE) observational study, and the retrospective Medicare-based analysis [7-9]. Nevertheless, to the best of our knowledge, the present study reports the largest cohort treated with XELOX/bevacizumab combination so far.

The age structure of patients in our study was similar to that reported in the NO16966, BRiTE, and BEAT studies. The present analysis used similar approach and was of comparable size as the Medicare analysis that, however, only included patients aged 65 year or older and only those with synchronous metastases [9]. This patient profile in the Medicare cohort may explain the difference in OS of almost 12 months compared to our study. The proportion of patients previously receiving adjuvant treatment was lower in the present study compared to the BEAT and BRiTE cohorts. Although the median PFS of patients in our cohort was similar to that reported in the above studies, the median OS was substantially longer, reaching 30 months for patients in the XELOX/bevacizumab subgroup. The favourable survival may be due to a more recent patient cohort in the present study and, possibly, patient selection. Gradual incremental improvements in OS have been observed in mCRC over the past decade because of the introduction of novel drugs and therapeutic strategies [10]. Importantly, the administration of the most expensive cancer drugs including bevacizumab has been centralised in the Czech Republic. It is possible that the centralisation of patients into cancer centres is partly responsible for the excellent survival results. In contrast, the median number of patients enrolled during a 16-month period per centre was only eight for the 248 sites participating in the BRiTE observational study [8].

For obvious reasons, the present analysis does not answer the question of the benefit of adding bevacizumab to an oxaliplatin-based regimen. The addition of bevacizumab has been shown to prolong OS in patients with irinotecan-based regimens, but the data on patients treated with the combination chemotherapy containing oxaliplatin are more ambiguous. In the NO 16966 trial that randomized patients using a 2 × 2 factorial design between XELOX and FOLFOX4 with or without bevacizumab, the addition of bevacizumab significantly prolonged PFS. However, statistically significant superiority could be demonstrated only in the subgroup of patients treated with XELOX but not FOLFOX. Adding bevacizumab to oxaliplatin-based chemotherapy resulted in a trend to prolongation of OS that did not reach statistical significance [2,11]. The lack of survival improvement after adding bevacizumab to oxaliplatin-based chemotherapy that contrasted with a significant effect in patients treated with irinotecan-based regimens has also been reported in the retrospective Medicare analysis [9].

The prognostic factors identified in the present cohort are well established for the mCRC population. The risk of death was almost two times higher in patients with three or more metastatic sites at the start of bevacizumab therapy compared to patients with only one metastatic site. The presence of two metastatic sites was associated with almost 50% increase in the risk of death. The risk of death was 34% higher in patients with metastatic CRC at diagnosis compared to patients with recurrent disease.

Of note, while the overall response rates in the FOLFOX/bevacizumab and XELOX/bevacizumab cohorts were similar, in the FOLFOX/bevacizumab group, there were significantly less patients who had disease stabilisation (31.4% versus 40.5%, respectively) and conversely more patients who had progressive disease than in the XELOX/bevacizumab group (15.0% versus 6.5%, respectively).

The possible differential association between OS and KRAS status for the two backbone regimens that reached borderline statistical significance in our analysis is surprising. We found that there was a trend to improved survival in patients with KRAS wild-type tumours versus those with KRAS mutated tumours in the XELOX/bevacizumab subgroup but in the FOLFOX/bevacizumab subgroup. This finding may merit further research.

Patients who had received prior adjuvant chemotherapy treated within the NO 16966 trial had better PFS with FOLFOX alone than with FOLFOX/bevacizumab [5]. We have carried out a similar analysis on our dataset but detected no statistically significant survival differences between the two studied combinations for any clinically defined patient subgroup (Table 4).

The present analysis has obviously several weaknesses that are partly due to its retrospective nature. Selection bias cannot be excluded as fitter patients could have been preferentially allocated to XELOX chemotherapy. Data on initial performance status are missing in one-half of the patients and there is some imbalance in the proportion of primarily metastatic patients between the cohorts. The registry does not provide data on the removal of primary tumours in patients with primarily metastatic colorectal cancer and on variant FOLFOX regimens that may be used in some centres, although these variables would be unlikely to skew the results of survival analysis.

No centralised review of radiological response was performed and the data on PFS may be less reliable given the number of centres involved and different patterns of care, including radiological imaging, in each centre. We were not able to extract valid data on metastasectomies from the registry for the entire period of study. On the other hand, the survival data from the registry were checked against the national registry of deaths. In general, OS data are more reliable for this type of registry-based retrospective studies, and some studies, including the study recently published by Meyerhardt *et al*., analysed only OS [9].

The incidence of adverse events was lower in the present analysis than that reported in prospective trials. The registry was focused on bevacizumab and the attending physicians apparently tended to report only events associated with bevacizumab and not toxicities linked to the chemotherapy backbone. Also, asymptomatic thrombotic events detected only on imaging, such as visceral thrombosis were unlikely to be reported. Because the incidence of severe or even life-threatening toxicities such as thromboembolism or gastrointestinal perforation that usually lead to treatment interruption or modification is less likely to be affected by underreporting, only grade 3–5 adverse events are reported here. Another reason for the relatively low incidence of adverse events could be the selection bias that is inherent to registry-based studies.

On the other hand, the strong point of the present analysis is that it shows similar activity of the combination of bevacizumab and FOLFOX/XELOX chemotherapies in real-world medicine.

## Conclusions

Data from a large, registry-based retrospective analysis suggest that XELOX and FOLFOX regimens in combination with bevacizumab are equipotent in the first-line treatment of mCRC. In a multivariable model, the number of metastatic sites was identified as the most significant predictor of PFS and, together with the presence/absence of metastatic disease at diagnosis, also for OS.

## Abbreviations

CRC: Colorectal cancer; mCRC: Metastatic colorectal cancer; OS: Overall survival; PFS: Progression-free survival; 5FU: 5-fluorouracil; IFL: Irinotecan, 5-fluorouracil, and leucovorin chemotherapy; FOLFOX: Infusional 5-fluorouracil, oxaliplatin and leucovorin chemotherapy; XELOX: Capecitabine and oxaliplatin chemotherapy; BEAT: Bevacizumab Expanded Access Trial; BRiTE: Bevacizumab Regimens’ Investigation of Treatment Effects observational study.

## Competing interests

TB has received speakers’ honoraria from Roche. TP has received speakers’ honoraria from Sanofi-Aventis. BM and IK have received speakers’ honoraria and have acted on advisory board for Roche.

## Authors’ contribution

TB, BM, and RO designed the study, performed the data analysis, and wrote the first draft. TP, ZB, and LD processed the data from the database and carried out statistical analysis. ZU, IK, MK, VB, RV, and JA acquired the data, interpreted the results, and co/wrote the manuscript. All authors have read, edited, and approved the final manuscript.

## Pre-publication history

The pre-publication history for this paper can be accessed here:

http://www.biomedcentral.com/1471-2407/14/323/prepub
